# The association between fecal microbiota, age and endoparasitism in adult alpacas

**DOI:** 10.1371/journal.pone.0272556

**Published:** 2022-08-25

**Authors:** Daniela Bedenice, Jessica Resnick-Sousa, Lauren Bookbinder, Victoria Trautwein, Hannah N. Creasey, Giovanni Widmer

**Affiliations:** 1 Department of Clinical Sciences, Cummings School of Veterinary Medicine at Tufts University, North Grafton, MA, United States of America; 2 Department of Infectious Diseases and Global Health, Cummings School of Veterinary Medicine at Tufts University, North Grafton, MA, United States of America; INRAE, FRANCE

## Abstract

Endoparasitism is a major cause of morbidity and mortality in alpacas (*Lama pacos*), with growing emergence of anthelmintic resistance. The purpose of the study was to correlate nematode worm burden and selected host phenotypic characteristics, such as age and weight, with the composition of the intestinal microbiota of adult alpacas. Fecal samples were collected per rectum from 102 healthy adult (2.1–11.2 years) alpacas at 3 separate timepoints (pre- and post-treatment with 8.8 mg/kg oral Levamisole HCL, and 4.6 months later) at a single farm. The profile of the fecal bacterial microbiota was characterized using 16S amplicon sequencing. Serial clinical exams and fecal egg counts were compared using related-samples analyses. The fecal microbiota of identically managed, healthy alpacas was characterized by a high level of temporal stability, as both α and β-diversity significantly correlated between sampling timepoints. Pairwise β-diversity between samples collected at each timepoint was low, ranging from 0.16–0.21 UniFrac distance units. The intensity of strongylid nematode infection (including *Haemonchus*, *Ostertagia*, *Trichostrongylus*) was only significantly correlated with microbiota composition in samples collected 14 days after treatment with levamisole. Analysis of similarity revealed no clustering of microbiota from anthelmintic responders or non-responders. Alpaca age explained the largest proportion of fecal microbiota variation and was the only consistently significant predictor of fecal microbiota taxonomic composition, by impacting the ratio of relative Bacteroidetes and Firmicutes abundance. Firmicutes, mostly Clostridiales, was the most abundant taxon across all collections.

## Introduction

Alpacas (*Lama pacos*), a domesticated species of South American camelids, contribute significantly to the non-food-producing US livestock population, with approximately 193,000 alpacas currently registered in the United States and an unknown number of unregistered animals [1]. Parasite management has become one of the most important concerns in the camelid industry, as endoparasitism is a major cause of morbidity and mortality in alpacas, with progressive development of anthelmintic resistance. The intensive use of anthelmintics to control gastrointestinal nematodes in small ruminants has led to a global emergence of anthelmintic-resistant nematode populations [2–4]. In fact, parasite resistance to all three major anthelmintic classes has been demonstrated worldwide in sheep and goats, over decades [5–8]. Similar resistance is also observed in alpacas [9, 10]. Anthelmintic resistance to fenbendazole, ivermectin, moxidectin and closantel in alpacas was recently reported [11]. It is therefore paramount to investigate non-pharmaceutical options to combat anthelminthic drug resistance and harness the gut microbiome as a resource for discovering novel therapeutical approaches and guiding responsible drug administration [12].

Little information is available on the intestinal microbial population of alpacas. A single study investigated the effect of diet on the microbiota in forestomach compartment 1 (C1), in the small intestine and in the large intestine of a very small number of alpacas [13]. While hay type (grass hay compared to alfalfa hay) significantly impacted gut microbiota composition in each body site, grain supplementation (approximately 25% of dry matter) over 2 weeks was insufficient to significantly alter the C1 microbiota in a second experiment. Forage-associated differences in microbial populations were primarily attributable to site-specific shifts in Bacteroidaceae, Prevotella and Actinobacteria. The dominant phyla (Firmicutes and Bacteroidetes [13]) identified in the analyses of alpacas were also found to be abundant in previous studies of ruminants and pseudoruminants, although most prior work was focused on C1 microbiota composition [14–16].

Emergent research suggests that the gut microbiome plays a role in either promoting or diminishing intestinal helminthic infection through microbe-driven changes to the gastrointestinal ecosystem and alterations in both innate and adaptive immunity [12, 17]. For example, gut microbiota are known to affect mucin biosynthesis and turnover. This effect is potentially important to host defense because the mucus layer inhibits the attachment of enteric pathogens to the epithelium. Additionally, microbiota mediate intestinal cell turnover and repair, prime protective innate immune responses or can create a pro-inflammatory environment that may increase helminth chronicity. Interactions between parasites and intestinal microbiota can thus alter the success of parasitic colonization, replication and virulence, and could critically alter infection outcomes and affect health [12, 17]. The purpose of the current study was to gain new insights into the composition of the healthy gut microbiota of alpacas, its response to endoparasites and association with selected clinical variables. Understanding the interaction between the gut microbiota and enteropathogens will facilitate the development of alternative, non-pharmaceutical treatments to increase resistance to enteric infections.

## Methods

### Field work and parasitology

The research was approved by the Tufts University Animal Care and Use Committee (project number G2021-78). One hundred and two clinically healthy, adult Huacaya alpacas were selected at a single farm located in New York state, based on a comparable pregnancy status, and exposure to an identical diet and farm management. The latter selection criteria were used to determine whether parasite burden or selected phenotypic properties contribute to differences in the fecal microbiota of healthy, pregnant alpacas. Prior to fecal collection, a complete diet history, medical history, and physical examination were obtained to ensure clinical health. Exclusion criteria included any recent gastrointestinal illness (colic, diarrhea), transport, medical treatment, dietary supplementation with probiotics or recent deworming. Age, body condition score (BCS), heart rate, respiratory rate, rectal temperature, attitude, and mucous membrane coloration were recorded prior to feces collection. During each sample collection timepoint, a blood sample was obtained via direct venipuncture using a 20 g one inch needle, for stall-side analysis of Packed Cell Volume (PCV, %) and Total Solids (TS, g/dL).

At total of 102 pregnant female Hucaya alpacas (median age: 4.4 years, range 2.1–11.2) were sampled before (timepoint 1; T1) and 2 weeks after deworming with 8.8 mg/kg Levamisole HCl (timepoint 2, T2). At the time of deworming (T1) the alpacas’ mean gestation was 116 ± 36 days. One hundred and 40 days later (timepoint 3; T3), a third sample was collected from 99 of 102 (97%) alpacas. Subsequently, 89 of 99 alpacas carried a pregnancy to term, with 83/89 (93.3%) live-births reported. Five alpacas remained open (pregnancy loss or non-pregnant), and 5 dams left the herd before parturition (outcome unknown). All alpacas were maintained together in a single group throughout the study period under identical housing conditions, receiving second cut free-choice hay (approximately 2% body weight) and 9 hours pasture turnout per day (5 alpacas/acre). Physical exam and laboratory monitoring performed at 3 separate timepoints are specified in S1 Table.

Fecal consistency was described on a scale of 0–5 according to the following guidelines: 0—Normal, firm separate fecal pellets; 1 –Formed pellets of feces that clump together and distort their shape with digital pressure; 2 –Soft fecal consistency where pellets mold together in a clump; 3—Pudding-consistency feces that still holds shape upon reaching the ground; 4—Pudding-consistency feces that spreads out upon reaching the ground; 5—Watery feces. Up to 20 mL of feces were collected per rectum using a clean gloved hand at three separate timepoints (pre- and post-treatment with 8.8 mg/kg oral Levamisole HCl, and 4 months later), for quantitative fecal egg count (FEC) analysis and for high-throughput DNA sequencing. All samples for FEC analysis were maintained on wet ice until processing within 36 hours, while samples for microbiota sequencing were immediately transferred on dry ice (for up to 12 hours) and then maintained at -80°C until DNA was extracted. The experimental design and methods were approved by the Clinical Studies Review Committee and informed client consent was obtained for all alpacas.

A quantitative FEC was performed for all animals within 24 hours of sample collection, using a modified centrifugation method [18]. Briefly, OvaSol Fecal Flotation Medium (Vedco Inc.) was reconstituted per manufacturer’s instructions the night prior to fecal testing, and the specific gravity verified to be 1.18 with a hydrometer. Five grams of feces were mixed with 15 mL OvaSol to create a slurry that was strained through a wire mesh strainer lined with gauze. The recovered liquid portion of the slurry was poured into a 15 mL polystyrene conical tube and spun in a non-refrigerated centrifuge at 1200 rpm (280 x g) for 10 minutes. The centrifuged mixture was topped off with OvaSol, dispensed against the sidewall of the test tube, until a convex meniscus was formed. A coverslip was placed on the meniscus and left for at least 10 minutes to incubate at room temperature, before being examined microscopically. All ova were counted and identified using a 10x as well as 40x objective lens to enhance classification as needed. The total ova count per gram feces was determined by the following formula: [(All ova counted) x (T / V)] / grams feces used, where T refers to the total volume of feces/flotation solution mixture and V to the volume of aliquot examined. Parasites were classified as strongylid eggs (including *Haemonchus*, *Ostertagia*, *Trichostrongylus*), *Nematodirus*, *Strongyloides*, *Eimeria*, *Giardia*, *Trichuris*, and other.

Following initial sample collection (timepoint 1) all alpacas were treated with 8.8 mg/kg oral Levamisole HCl (equivalent to 7.62 mg/kg of levamisole; Prohibit Drench Powder^TM^) according to manufacturer’s instructions, using a commercial dose syringe (Duratek^TM^ Dosing Syringe) to administer medications to the back of each alpaca’s mouth under light manual restraint. Levamisole was expected to be effective against strongylid nematodes based on prior results of a commercial *in vitro* Larval Development Assay (DrenchRite^TM^) performed for clinical purposes as previously described (University of Georgia Veterinary Diagnostic Laboratory, Athens GA) [19]. The effectiveness of levamisole was determined by estimating the reduction in fecal egg count (FEC) between pretreatment (sampling timepoint 1) and 14 days after treatment (timepoint 2). The treatment was considered effective for an animal if a FEC reduction ≥95% was observed [20]. In the current study, alpacas were classified as consistent strongylid egg shedders if they excreted ≥300 epg (eggs per gram) feces at 2 of the 3 timepoints, and as high shedders if the FEC exceeded 600 strongylid epg at any single timepoint.

### Clinical data analysis

Clinical data are presented descriptively as mean +/- standard deviation (SD) or median +/- interquartile range (IQR) or range. Univariate statistical analyses were based on the normality of data distribution (Shapiro-Wilk test), employing correlation analyses (Pearson Correlation or Spearman’s rho), Related-Samples Friedman’s Two-Way Analysis of Variance, paired samples T-Test, McNemar’s Chi-Squared, Mann-Whitney U, and Wilcoxon Signed Rank Tests to assess animal data and sampling timepoints, with an accepted significance level of P<0.05. These numerical analyses were performed with the IBM SPSS Statistics 26 package.

### Microbiota and bioinformatics

DNA was extracted from 200 μL of feces in a Qiacube instrument using the QIAamp PowerFecal DNA kit according to the manufacturer’s instructions. Fecal DNA was eluted in 50 μL elution buffer and stored at -20°C. A 2-step PCR protocol described previously [21] was used to amplify and barcode the V1V2 16S rRNA variable region. Up to 106 barcoded amplicons were pooled in approximately equal molar proportion. The size-selected library was sequenced in an Illumina MiSeq instrument operated by Tufts University genomics core facility (tucf.org). A total of 317 amplicons were sequenced and 29.7 x 10^6^ unscreened and unfiltered sequences were obtained. To control for technical variation, each library included duplicates of two randomly chosen samples. Duplicated amplicons were amplified from two DNA samples extracted in parallel from the same fecal samples. Each duplicated amplicon was tagged with a unique barcode. An amplicon generated from a synthetic bacterial population (BEI Resources, Manassas, VA, cat no. HM-782D) was also included in the library as quality control. Because libraries from the three collections (timepoints) were sequenced in separate MiSeq runs, 2 randomly selected samples from each library were re-sequenced in a subsequent library to control for a possible library effect. The mean UniFrac distance [22] between these replicated samples was 0.08 (SD = 0.006, n = 6), demonstrating the absence of such an effect.

FASTQ formatted sequence files were processed primarily using programs in *mothur* [23] essentially as described [21]. Prior to any screening and filtering, an average of 93,746 sequences reads were obtained per barcode (n = 317 barcodes, SD = 52,958). The mean quality score for the entire sequence collection was 32.3 (SD = 1.58). Each sample was randomly subsampled to 5000 sequences. Subsamples were curated by removing sequences that did not align, sequences containing ambiguous base calls or homopolymers longer than 8 nucleotides and sequences shorter than 210 nucleotides (nt). Singletons differing by 1 SNP from the most similar neighbor were removed using program *pre*.*cluster* [23]. After curation, an average of 2808 sequences/sample were retained for downstream analysis.

Pairwise β-diversity between samples was quantified in *mothur* [23] using the weighted UniFrac distance metric [22]. To keep the computational effort manageable, analyses combining samples for the 3 timepoints were limited to 40 randomly chosen alpacas. Phylip-formatted distance matrices were imported into GenAlEx [24] and visualized using Principal Coordinate Analysis (PCoA). Operational Taxonomic Units were created in *mothur* using the Opticlust method [25] and a sequence dissimilarity cutoff of 0.03%. OTUs with average relative abundance in each collection of <1 sequence/sample were removed using program *remove*.*rare* [23]. Ordination analyses other than PCoA were performed in CANOCO 5 [26]. Redundancy analysis (RDA) [27] was applied to associate the independent variables such as strongylid epg, consistent shedder status, age and weight with OTU profile. In this analysis, the statistical significance of any association is evaluated by permuting the samples in the OTU table with respect to the independent variables [26]. Sequences were taxonomically classified using *classify*.*seqs* in *mothur* [23]. Template and taxonomy files (version 132) with 213,126 sequences x 50,000 columns were downloaded from SILVA [28]. A 75% probability cut-off was applied. Linear Discriminant Analysis was performed in *mothur* using program *LefSe* [29].

## Results

### Clinical observations

Sixty-three % (64/102) of alpacas were positive for endoparasites prior to deworming upon initial sample collection. At this time, strongylid nematode infection predominated with 44/102 (43.1%) affected alpacas (median positive egg count: 300 epg, range: 150–4200 epg). Thirty-four % (35/102) of alpacas remained fecal positive 2 weeks after deworming with levamisole, with 11/102 (10.8%) retaining evidence of strongylid nematode infection. A 100% reduction in strongylid FEC was achieved after deworming in 35/44 (80%) positive alpacas at 2 weeks, with a ≤50% FEC reduction observed in the remaining 9 animals, and 2 alpacas showing new strongylid nematode infections. Overall, 22/102 (21.6%) alpacas were considered consistent strongylid egg shedders (FEC ≥300 epg at more than one time point) across 3 collection periods, and 35/102 (34.3%) were classified as high shedders (FEC ≥600 strongylid epg). Complete fecal results are listed per timepoint in Table 1.

**Table 1:** Fecal egg counts of 102 clinically healthy alpacas*.

Variable	Timepoint 1	Timepoint 2	Timepoint 3
FEC** positive (%)	64/102 (62.7%)^1^	35/102 (34.3%)^2^	70/99 (70.7%)^1^
Strongylid egg positive (%)	44/102 (43.1%)^1^	11/102 (10.8%)^2^	58/99 (58.6%)^3^
*Eimeria* positive (%)	23/102 (22.5%)^1^	19/102 (18.6%)^1^	36/99 (36.4%)^2^
*Strongyloides* positive (%)	11/102 (10.8%)^1^	3/102 (2.9%)^2^	2/99 (2%)^2,3^
Strongylid epg (median, IQR)	300 (600)	150 (150)	600 (1088)
*Eimeria* opg (median, IQR)	300 (300)	300 (450)	300 (450)
Strongyloides epg (median, IQR)	150 (150)	450 (-)	150 (0)

*Related-Samples McNemar’s Chi-squared test: Within each row, different numbers in superscript indicate significant differences between timepoints, P<0.05.

**FEC, Fecal Egg Count; opg, oocysts/gram feces; epg, eggs/g feces; IQR, inter-quartile-range

### Microbiota analysis

#### 1. Analysis of individual timepoints

**Fig 1:**
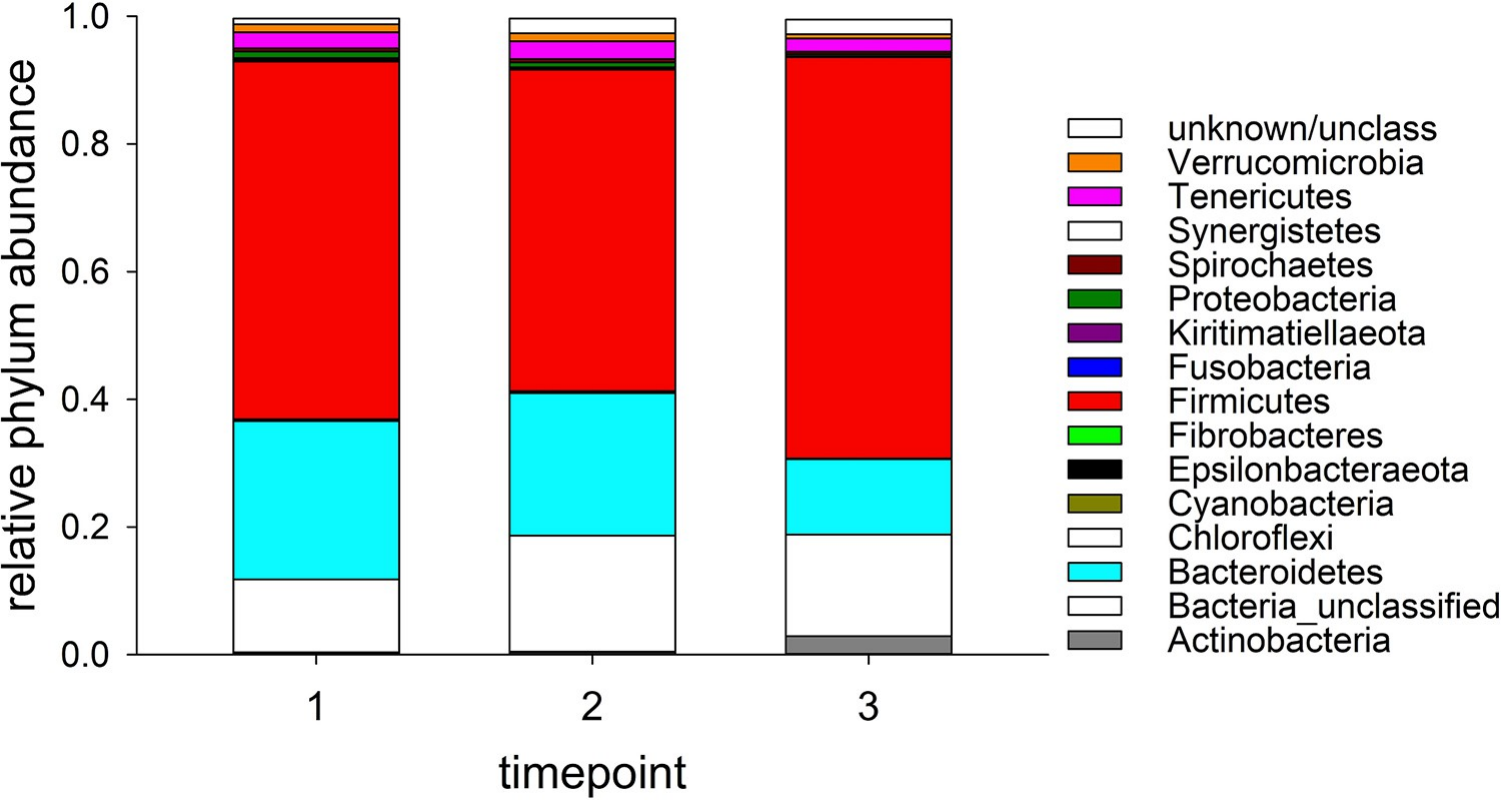
Classification of alpaca fecal microbiota 16S sequences reveals a high relative abundance of Firmicutes. The analysis is based on 258,889 unique curated 250-nt sequences, used the method of Wang [30] and has a confidence threshold of 75%.

Fig 1 shows the average phylum-level taxonomy for each timepoint. Firmicutes, mostly Clostridiales, was the most abundant phylum (order), representing more than half of all classified sequences. Bacteroidetes represented 25%, 22% and 11% of all sequences at timepoint 1, 2 and 3, respectively. The complete taxonomy by timepoint in shown in S2 Table. Weighted UniFrac distances between samples collected at the same timepoint were generally very low, averaging 0.16, 0.16 and 0.21 for the first, second and third timepoint, respectively. We verified that low α diversity was not an artefact of sequencing depth (S1 Fig in S1 File).

To identify clinical variables significantly associated with microbiota profile, RDA was applied by defining alpaca age, strongylid egg shedder status (>300 eggs in 2/3 samples) and weight as explanatory variables. Of the 3 variables, only age was consistently associated with the OTU profile (Table 2). Whereas this association was significant at all timepoints, the fraction of OTU variation explained by age, weight and strongylid epg was low: 3.0% for timepoint 1, 4.9% for timepoint 2 and 7.3% for timepoint 3.

**Table 2:** Association of 3 clinical variables with microbiota profile.

			independent variables		
timepoint	number of samples	number of OTUs	age	Strongylid (epg)	consistent shedder****
1	102*	698	F** = 3.2, p = 0.0001	F = 1.2, p = 0.09	R*** = 0.11, p = 0.055
2	96*	520	F = 2.5, p = 0.002	F = 2.5, p = 0.002	R = 0.25, p<0.001
3	98*	531	F = 4.8, p = 0.0001	F = 0.9, p = 0.59	R = 0.08, p = 0.106

* OTU abundances in duplicated samples were averaged.

** pseudo-F calculated according to ter Braak and Šmilauer [26]

*** ANOSIM R calculated from UniFrac β diversity values [31]

**** >300 strongylid epg in 2/3 samples.

Because age and weight are correlated in this herd, (timepoint 1: Pearson r = 0.38, p = 8.2 x 10^−5^; timepoint 2: r = 0.37, p = 2.4 x 10^−4^; timepoint 3: r = 0.32, p = 6.7 x 10^−3^), we tested whether the association between age and OTU profile remained significant after removing the effect of weight from the analysis. To this aim, weight was defined as covariate and age as independent variable. In this RDA, age continued to be significantly associated with the OTU profile (timepoint 1, pseudo-F = 2.9, p = 0.0001; timepoint 2, pseudo-F = 2.2, p = 0.002; timepoint 3, pseudo-F = 2.9, p = 0.0001), demonstrating the importance of age in shaping the fecal microbiota. The analysis of worm burden based on strongylid egg concentration and persistent shedder status returned consistent results in spite of the two analyses being based on a continuous and a categorical variable, respectively. Both analyses concur in finding only fecal microbiota from timepoint 2 significantly associated with worm burden (S2 Fig in S1 File). Whether this outcome is related to treatment with levamisole is unknown because all animals were dewormed. LDA was applied to identify bacterial taxa explaining the difference in microbiota profile between timepoint 2 samples from alpacas classified as consistent shedders (n = 21) and those not belonging to this group (n = 75). LDA identified 40 OTUs out of 520 OTUs significantly associated with shedding status (S3 Table).

To identify bacterial taxa significantly associated in relative abundance with alpaca age, OTUs were ranked according to their linear dependence on age. For this analysis, the RDA OTU score on ordination axis 1, denoted *Resp*.*1* in CANOCO [26], was used as a measure of linear dependence of the relative abundance of each OTU on age, where age was defined as the independent variable. The phylum-level taxonomic classifications of the 30 OTUs with the most positive and the most negative regression coefficient were entered into a contingency table to test the statistical significance of the association between taxonomy and *Resp*.*1*. A Chi-square test returned a highly significant association between these variables for each of the three timepoints if the OTU counts were weighed according to the number of sequences assigned to each OTU (Table 3). The Chi-square analysis for the unweighted OTU data was significant for timepoint 1 and 3, but not for timepoint 2. Although these results show that age is a significant predictor of OTU profile, the actual taxonomy of the OTUs which most closely correlate with age is surprisingly diverse. At timepoints 1 and 2, most OTUs with a strong positive association with alpaca age are classified as *Bacteroidetes*, mostly in the order *Bacteriodales*. In contrast, at timepoint 3, the 30 OTUs most positively associated with age were Firmicutes, mostly in the class *Clostridia*. At the negative end of the regression coefficient range, *Bacteroidetes* was the most abundant phylum at all timepoints, and *Bacteriodales* was the most abundant order. This is shown in Table 3 on the second row for each timepoint. S4 Table shows the taxonomy of the 10 most abundant OTUs for each animal and timepoint, together with selected clinical variables.

**Table 3:** Taxa significantly associated with age*.

timepoint	Resp.1**	Bacteroidetes	Firmicutes	Other classifications***	Chi-square 2 d.f.****
1	>0.320	6104 (78%)	1514 (19%)	242 (3%)	1506 p<0.001
	<-0.243	5445 (55%)	4440 (45%)	0	
2	>0.257	4795 (57%)	2473 (30%)	1073 (13%)	18 p<0.001
	<-0.246	4426 (56%)	2298 (29%)	1202 (15%)	
3	>0.300	0	16864 (97%)	528 (3%)	14893 p<0.001
	<-0.355	5298 (60%)	2728 (31%)	762 (9%)	

*Analysis based on RDA with age as sole independent variable; shown are counts of sequence reads and % of row total.

** Regression coefficient of 30 most linearly dependent OTUs with respect to age. Each row refers to 30 OTUs. The regression coefficient values shown in column Resp.1 indicate the cut-off.

*** Includes unclassified

**** Weighed by number of sequences assigned to each OTU

In terms of levamisole efficacy, 35/44 animals with strongylid nematode infection showed a 100% reduction in strongylid epg between timepoint 1 and timepoint 2 (treatment responders), while the remaining 9 alpacas were considered non-responders (≤50% FEC reduction). ANOSIM [31] was used to test whether the microbiota in responders and in non-responders was different. This analysis revealed no significant clustering of responders and non-responders’ microbiota (R = -0.22, p = 0.55).

#### 2. Comparisons across collections

To exclude possible library effects on comparisons between samples sequenced in different libraries, we included the same amplicons in different libraries and calculated the weighted UniFrac distance between these replicates. Consistent with the absence of an effect of the library or the sequence output, the mean of 5 UniFrac distances between replicates was 0.080 (SD = 0.006), well below the average within-library β diversity of 0.16 (timepoint 1), 0.16 (timepoint 2) and 0.21 (timepoint 3). As a second quality control, we compared β diversity between pairs of samples sequenced in the same reaction. For instance, the 16S amplicons of alpaca 2582 and 2643 from the third collection were both sequenced in two libraries, generating 4 sets of sequence reads. This design was intended as an additional quality control to detect possible library/sequencing artefacts. For three sample pairs (n = 6) included in this QC, the weighted UniFrac values were very similar across libraries, i.e., 0.1089 vs. 0.1166, 0.1262 vs. 0.1334 and 0.1339 vs. 0.1555. We concluded that the sequence profiles were not affected by the sequencing reaction, supporting an integrated analysis of samples from the 3 timepoints described in this section.

To assess the magnitude of microbiota changes over the 3 timepoints, β-diversity between samples collected from the same 40 alpacas at different timepoints was computed using UniFrac distance. β diversity between timepoint 1 and 2 was 0.15 (n = 40, SD = 0.02), between timepoint 1 and 3 was 0.29 (n = 40, SD = 0.05) and between timepoint 2 and 3 was 0.28 (n = 40, SD = 0.05). These values correlate with the time between collections in the sense that longer time periods between sample collections are associated with a larger mean UniFrac distance. The small diversity between pre- and post-Levamisole treatment are indicative of a lack of drug effect on the microbiota.

**Fig 2:**
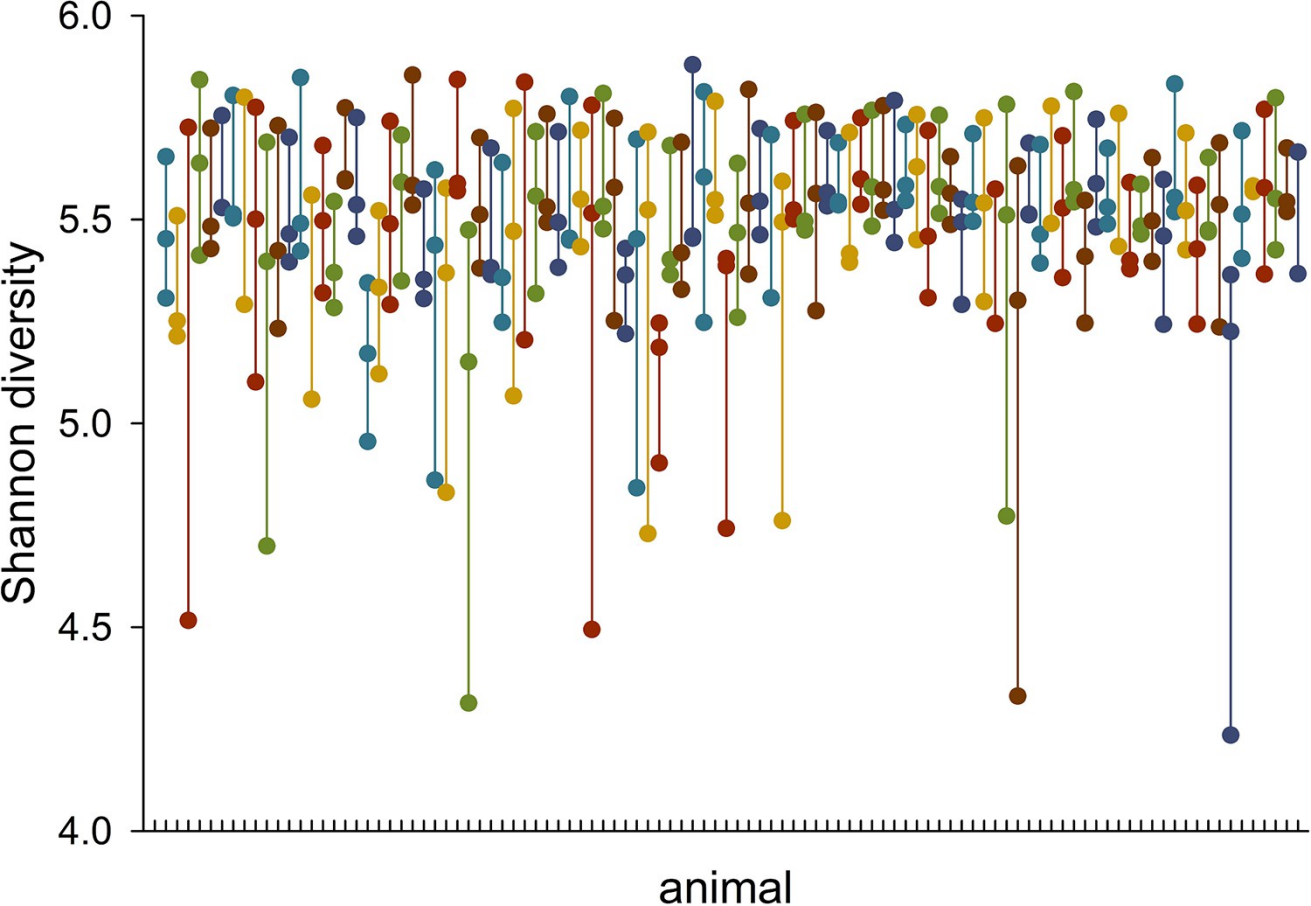
Persistence of low- and high-diversity microbiota over the 4-month study period. The Shannon diversity values from each animal’s microbiota are aligned vertically and connected by a line. Colors have no meaning other than improving visualization.

**Fig 3:**
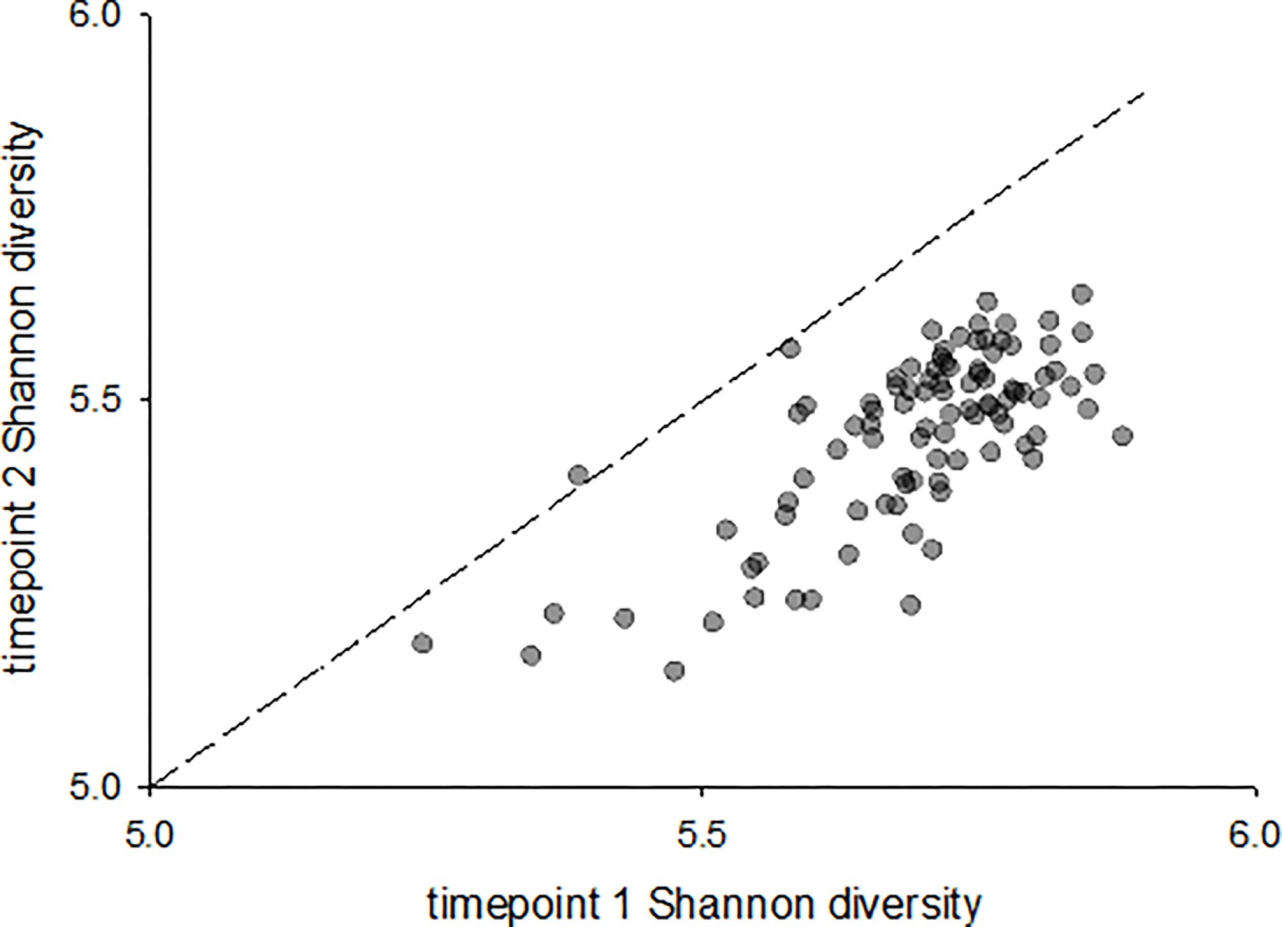
Shannon diversity is correlated across collections. Each datapoint represents one animal. Shannon diversity for the first and second timepoint from each animal is plotted on the x and y axis, respectively. y = x line is shown to visualize drop in α diversity between the two timepoints.

Mean microbiota α diversity was 5.7 (n = 102, SD = 0.11), 5.4 (n = 96, SD = 0.11) and 5.3 (n = 98, SD = 0.31) for timepoint 1, 2 and 3, respectively. Alpacas harboring a fecal microbiota with low α diversity at one timepoint were likely to be populated with a similarly low-diversity microbiota at the other 2 timepoints (Table 4). This trend is illustrated in Figs 2 and 3.

**Table 4:** Fecal microbiota α diversity is highly correlated between collections.

	1	2	3
1		n = 96, p = 2.44E-017	n = 96, p<0.0001
2	0.732		n = 92, p = 0.0001
3	0.428	0.391	

Upper triangle: n, number of pairwise comparisons; p, type I error probability. Lower triangle: Pearson r.

**Fig 4:**
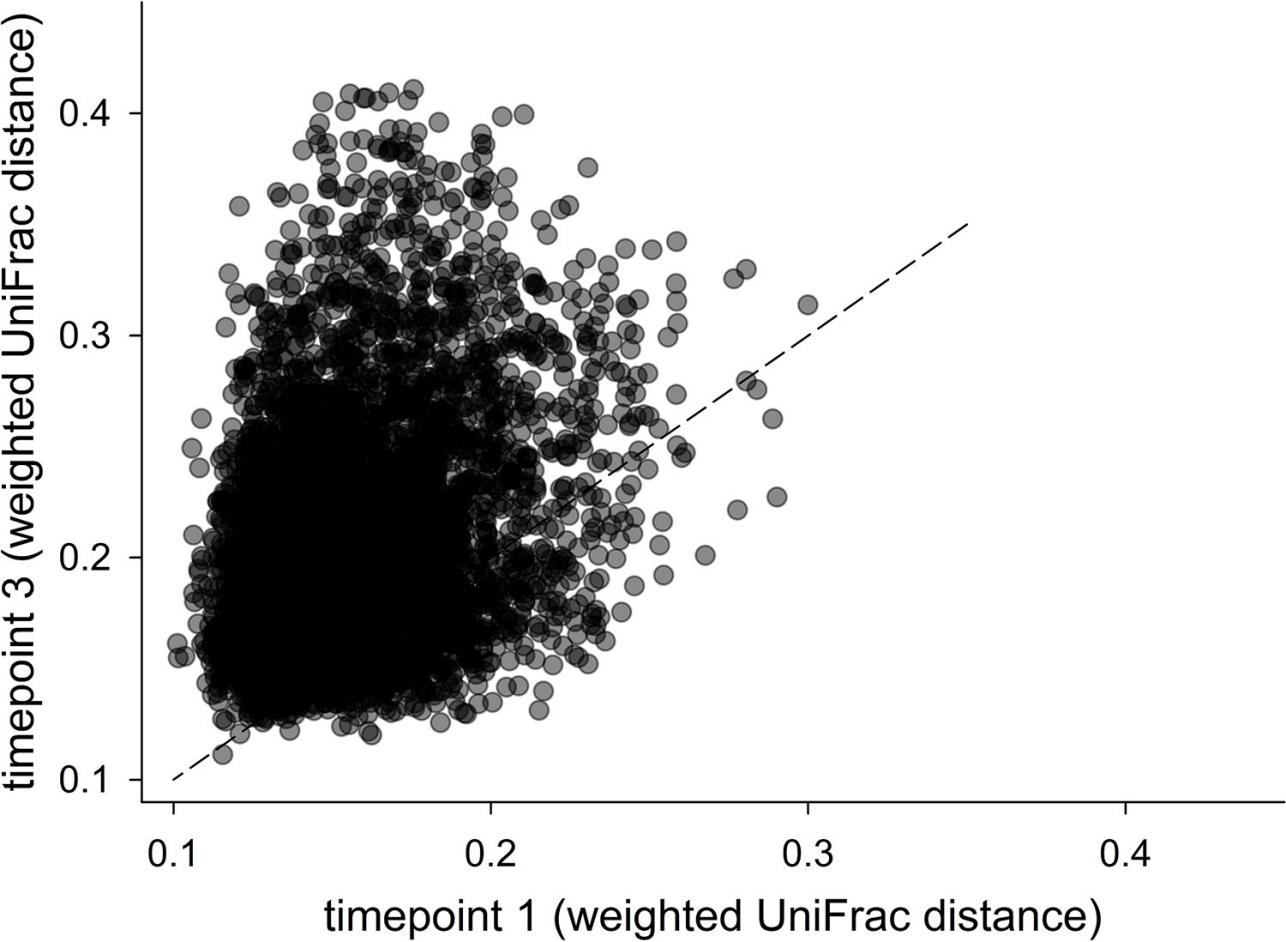
Pairwise UniFrac distances are positively correlated across collections. Each datapoint represents the β diversity between microbiota from two animals measured using weighted UniFrac distance. The plot illustrates the correlation between the β diversity observed in the first and third timepoint; Pearson correlation r = 303, n = 5246, p = 8.1E-112. Dashed line represent y = x.

As found with the comparison of α diversity values across timepoints, Fig 4 shows a similar trend for β diversity. The positive correlation summarized in Table 5 and illustrated in the figure demonstrates that animals populated by more distinct microbiota at one timepoint, were likely to harbor distinct microbiota at the other timepoints as well. Fig 4 also shows that, on average, β diversity was higher between samples collected on timepoint 3 as compared to the β diversity between samples from the first timepoint and between samples from timepoint 2 (timepoint 1 mean UniFrac distance = 0.16, timepoint 2 mean = 0.16, timepoint 3 mean = 0.21). This comparison shows that β diversity slightly, but significantly, increased over time (ANOVA on ranks, H = 3804, 2 d.f., p<0.001).

**Table 5:** Fecal microbiota β diversity is highly correlated between collections*.

	1	2	3
1		n = 4848, p = 3.1E-108	n = 5246, p = 8.1E-112
2	0.310		n = 4462, p = 1.2E-51
3	0.303	0.224	

*Upper triangle: n, number of pairwise comparisons, p, Pearson r statistical significance; lower triangle: Pearson r.

## Discussion

The analysis of alpaca fecal microbiota using 16S amplicon sequencing revealed a relative homogeneity between alpacas at all 3 timepoints (β diversity 0.16–0.21). This observation is consistent with the fact that the animals were healthy, subject to the same treatment, belonged to the same herd and were fed the same diet. The β diversity between microbiota from different alpacas is lower than observed among healthy horses from individual barns, using the same 16S protocol [32, 33] (S3 Fig in S1 File). Alpha diversity was also low compared to previously reported data from young-adult Bactrian camels, a species closely related to alpacas [34]. Since microbiota diversity in healthy, phenotypically similar animals is largely driven by diet and environment, it is likely that the identical management conditions for the alpacas analyzed in this study minimized β diversity. The impact of different sequencing strategies, such as the 16S variable region sequenced, should also be considered, particularly when comparing α diversity values from different surveys.

The original goal of this study was to assess whether the fecal microbiota of alpacas is impacted by enteric nematodes. The analyses reported here do not clearly answer this question as the intensity of strongylid nematode infection was only a significant predictor of fecal microbiota composition at timepoint 2, and only explained 2.5% of OTU variation. When grouping strongylid infected alpacas based on their response to levamisole treatment, ANOSIM revealed no clustering of microbiota from anthelmintic responders or non-responders. These observations do not support the view that levamisole-resistance of strongylid nematodes significantly impacted fecal microbial composition.

Anthelmintic resistance in parasites is widespread in camelids, similar to observations in small ruminants. Aggregated results of a recent European meta-analysis in sheep and goats identified an average prevalence of resistance to levamisole of 48% between 2010 and 2020, in comparison to 86% for benzimidazoles, 52% for macrocyclic lactones (except moxidectin), and to 21% for moxidectin. Reports of farms with gastro-intestinal nematodes resistant to levamisole became more common between 1980 and 2009, but then decreased between 2010–2020 [4]. Levamisole treatment was selected here based on farm-specific results of a prior DrenchRite^TM^ Larval Development Assay. Levamisole has been assigned to pregnancy category C by the FDA, since animal studies have revealed evidence of embryotoxicity. Treatment with immunomodulatory (2.5 mg/kg) doses of levamisole during conception or early gestation are reported to reduce pregnancy rates in ewes, although anthelmintic doses (7.5 mg/kg) did not disrupt establishment of pregnancy in the same study [35]. Since mean alpaca gestation was 116 ± 36 days at the time of levamisole treatment, adverse reproductive effects were not expected. Ultimately, 89 of 94 (95%) pregnant alpacas that remained at the farm until parturition carried a pregnancy to term with 93% live births reported.

Since experimental interventions were precluded in our study of healthy alpacas, the data do not support any analyses to assess the effect of anthelmintic treatment on fecal microbiota. Therefore, we could not determine whether the observed association between fecal microbiota and strongylid egg burden at timepoint 2 (two weeks after deworming) was related to levamisole treatment. However, a small, overall drop in α diversity was identified between the first (pre-treatment) and second collection (2 weeks post treatment) (Fig 3), similar to prior observations in horses after deworming. Shannon (α) diversity decreased in horses on days 2 and 7 after deworming with moxidectin plus praziquantel [36] and moxidectin or fenbendazole [37], respectively, and reversed by day 14 in the latter study. The high degree of similarity between microbiota from collection 1 and 2 in alpacas, nonetheless, indicates that levamisole had no major effect on the fecal microbiota, despite reaching a median 100% (mean 84%) strongylid egg reduction at timepoint 2. Likewise, a previous study in goats revealed that partially effective anthelmintic treatment (84% FEC reduction) with moxidectin did not affect indices of microbial diversity in feces collected from the rectal-anal junction. However, the abundance of at least two phyla, Proteobacteria and Planctomycetes, was significantly increased in the hind-gut after moxidectin treatment [38]. Since a disproportionally high Proteobacteria to Firmicutes ratio is documented in *Haemonchus* larvae, the authors proposed that surviving worms may have contributed to this phenomenon [39].

Consistent with the absence of disease, other than parasitic infections, the microbiota of the individual alpacas displayed a remarkable stability over time, as both α and β diversity tended to persist across timepoints. Nonetheless, timepoint 3 (spring) samples diverged more from timepoints 1 and 2 than these timepoints did from each other, leading to a slight but statistically significant increase in β diversity in the third sample collection. A possible reason for this trend is the availability of fresh grass in April. Diet changes in the early spring were related to the disappearance of the snow cover, giving animals more access to pasture grass around timepoint 3. Spring samples were not only more different from those collected in December (timepoints 1 and 2), but were also more divergent from each other, as demonstrated by higher β diversity values. The number of parasite-free alpacas did not differ between timepoint 1 (winter collection before deworming) and timepoint 3 (spring collection, Table 1), and OTU variations at T1 and T3 were unassociated with the intensity of strongylid nematode infection, supporting that a springtime rise in β diversity was unrelated to parasitism.

The alpacas enrolled in this study were in overall good clinical health, indicating that parasitic infections had a limited adverse impact on the animals. It is thus possible that parasitism leading to clinically illness in a different study population of diseased alpacas, would have been more likely to impact microbiota composition. However, clinically healthy animals were chosen to determine if shedder status, rather than clinical illness, shaped microbiota profiles. Additionally, it was not possible to determine if shedding of strongylid eggs was specifically associated with *Haemonchus*, *Ostertagia*, or *Trichostrongylus* infection, as the latter cannot be differentiated based on microscopic FEC analysis. Although *Haemonchus* was identified as the predominant nematode infection in several recent alpaca studies [10, 40], a coproculture was unavailable to estimate the proportion of each genus in the current study. Age was the only clinical parameter examined which was consistently associated with the OTU profile at all 3 timepoints, but explained less than 10% of fecal microbiota variation. In contrast, a prior equine study found that age did not affect the relative abundance of bacterial phyla in fecal microbiota of healthy young-adult (2–12 years) compared to geriatric horses (≥ 20 years) on comparable diets at the same barn [32]. Similarly, Dougal et al reported no differences in bacterial community structure between healthy adult (5–12 years) and elderly (19–28 years) horses [41]. In contrast, the relative abundance of several amplicon sequence variants changed with increasing age in healthy horses and ponies evaluated in the Netherlands [42]. In the present study, the actual taxonomy of OTUs which most closely correlated with age were classified as *Bacteroidetes* in the winter, and Firmicutes in the spring collection, and may have been influenced by diet, with an increasing availability of pasture grass by early April. Across all three timepoints, Firmicutes was the most abundant phylum found in the present analysis. This observation is in agreement with a prior report in camels [34]. The relative abundance of Bacteroides in our study is however clearly smaller than observed in a study of alpacas fed different diets [13]. Similar to camelids, Firmicutes is the main bacterial phylum identified in the fecal microbiota of most equine studies to date, while fewer reports identified Bacteroidetes as the most abundant phylum in horses [42]. A comparison of taxa abundance and microbiota composition between studies using different protocols remains challenging, as the effect of different 16S primers, DNA extraction methods and sample handling remains poorly characterized.

## Conclusions

The fecal microbiota of identically managed, healthy alpacas was characterized by a high level of temporal stability, as both α and β-diversity tended to persist across sampling timepoints. Firmicutes was the most abundant phylum across all collections, with alpaca age being identified as the only consistently significant predictor of fecal microbiota composition. A significant association between strongylid egg burden and fecal microbiota profile was only detected in one of three timepoints. Even in this sample, strongylid egg concentration explained a small proportion of microbiota variation.

## Supporting information

S1 File (DOCX)

S1 Table Clinical parameters of 102 healthy alpacas at three timepoints. (DOCX)

S2 Table Taxonomy by alpaca and by timepoint. (XLSX)

S3 Table LDA analysis of timepoint 2 samples grouped according to consistent shedder status. (XLSX)

S4 Table Ten most abundant OTUs, fecal egg count, by timepoint and alpaca including timepoint 1 age and weight. (XLSX)
