# Supplementary material for: The association between fecal microbiota, age and endoparasitism in adult alpacas
Source: PLoS One. 2022 Aug 25;17(8):e0272556. doi: 10.1371/journal.pone.0272556 (PMC9409599; doi:10.1371/journal.pone.0272556)
Supplement: S1 File — (DOCX) [file pone.0272556.s001.docx]

**Additional file 1. Supplemental figures.**

**Fig. S1.** Low α diversity is not an artefact of sequencing depth as microbiota with low diversity have average sequence depth. Each datapoint represents one alpaca. Data are from timepoint 3. Y axis represents z score where 1 unit = 1 standard deviation.

**Fig S2**. Principal Coordinate Analysis plot representing weighted UniFrac distance between fecal microbiota from 103 alpacas at timepoint 1. Shading is proportional to strongylid egg count, where white indicates no nematode eggs and black corresponds to 4200 epg, which was the highest count for this timepoint. Three samples were replicated to estimate technical variation. Matching triangle symbols and diamonds indicate replicates. Samples do not cluster based on strongylid FEC, indicating that β-diversity between samples at timepoint 1 was unrelated to parasite burden. The proportion of variation explained by each axis is indicated in percent. PC, Principal Coordinate.

**Fig. S3**. Pairwise weighted UniFrac distances between alpaca fecal microbiota collected at the same timepoint compared with distances between fecal microbiota previously collected from healthy horses. Since horses belonged to four different herds, designated N1-N4 in previous publications , only distances between horses stabled in the same location were included. This analysis revealed a significantly lower β diversity between alpacas than between horses (Mann-Whitney Rank Sum test, U=89500, p<0.001). The plot is based on 15178 and 570 weighted UniFrac distances for alpacas and horses, respectively. Horizonal line, median; box 25-75 percentile; whiskers 10-90th percentile.
