# Supplementary material for: The association between fecal microbiota, age and endoparasitism in adult alpacas
Source: PLoS One. 2022 Aug 25;17(8):e0272556. doi: 10.1371/journal.pone.0272556 (PMC9409599; doi:10.1371/journal.pone.0272556)
Supplement: S1 Table — (DOCX) [file pone.0272556.s002.docx]

**Additional file 2. Supplemental table**

**Table S1:** Relevant clinical parameters were compared between collection timepoints, to assess alpaca health throughout the study period. Although statistically significant variations in vital and laboratory parameters were observed in alpacas across collection timepoints, the magnitude of change was of limited clinical significance.

| **Clinical Variable**  Median (IQR) | **Timepoint 1** | **Timepoint 2** | **Timepoint 3** | **P value** |
| --- | --- | --- | --- | --- |
| Fecal score (out of 5) ^(c)^ | 0 (1)^1^ | 1 (1)^2^ | 0 (1)^1^ | <0.001 |
| Weight (lb) ^(a,b)^ | 148.7 (22.9)^1^ | 146.6 (22.7)^2^ | 150.6 (30.0)^3^ | <0.001 |
| Body Condition Score ^(c)^ | 3.0 (1.5) | – | 3.5 (1.4) | <0.001 |
| Heart Rate (bpm) ^(c)^ | 64 (14) | – | 56 (8) | <0.001 |
| Respiratory Rate (brpm) ^(c)^ | 20 (8) | – | 24 (8) | <0.001 |
| Temperature (°F) ^(c)^ | 100.7 (0.9)^1^ | – | 100.6 (1.1) | 0.111 |
| PCV (%) ^(b)^ | 30 (3)^1^ | 31 (4)^2^ | 31 (4)^2,3^ | <0.001 |
| TS (g/dL) ^(b)^ | 6.8 (0.5)^1^ | 6.8 (0.6)^2^ | 6.9 (0.5)^1,2^ | 0.011 |

**PCV,** Packed cell volume; **TS,** total serum solids; –, not assessed

^(a)^ mean (standard deviation)

^(b)^ Related-Samples Friedman's Two-Way Analysis of Variance: Within each row, different numbers in superscript indicate significant differences between timepoints

^(c)^ Related-Samples Wilcoxon Signed Rank Test
